# Lutein and Neurodevelopment in Preterm Infants

**DOI:** 10.3389/fnins.2016.00411

**Published:** 2016-09-07

**Authors:** Matteo Giampietri, Francesca Lorenzoni, Francesca Moscuzza, Antonio Boldrini, Paolo Ghirri

**Affiliations:** Neonatal Departement, S Chiara HospitalPisa, Italy

**Keywords:** lutein, preterm infants, neurodevelopment, epigenetic, carotenoids

Carotenoids are pigments naturally occurring in plants, particularly in dark-green leafy vegetables such as raw spinach and cooked kale, and they have many functions in human tissues. Lutein is the dominant carotenoid found in adult and in infant brain especially in the neocortex and in the neural retina, however diet intake is needed because it cannot be synthesized in the body (Costa et al., 2013). Preterm infants have been deprived from carotenoid transfer during the last weeks of pregnancy and after birth the only sources are breast milk or oral supplementation. Concerning carotenoids, it has long been assumed that their intestinal absorption occurs by passive diffusion. Recently the high inter-individual variability in absorption observed in human studies argues in favor of the existence of putative membrane transporters of carotenoids. After uptake by the enterocyte, a substantial quantity of carotenoids up to 40% of the dietary intake is not metabolized. It is assumed that the major fraction of carotenoids are incorporated into chylomicrons that are secreted into the lymph (Reboul, 2013). Carotenoid composition of human milk changes during the first month postpartum. Lutein represents only 20% of carotenoids in colostrum but nearly 50% in mature milk. During the first month postpartum all four carotenoids (lutein, β-cryptoxanthin, lycopene, and α-carotene) decrease in the milk but lutein remains relatively elevated probably because it plays an important role for the infant (Gossage et al., 2002). Lutein concentration in breast milk correlates with mother plasma concentration and lutein intake during the third trimester of pregnancy. There is a dose-dependent response to lutein supplementation in lactating women. Previous studies (Sherry et al., 2014) demonstrated that lutein supplementation in pregnant women increases breast milk lutein concentration and provides plasma increase in their infants. Mothers who received higher doses of lutein supplementation had higher value of lutein concentration in plasma than mothers who received lower doses or placebo. However arterial cord blood lutein levels in addition to maternal plasma concentrations seem to be sex, gestational age and delivery mode dependent. Lutein levels are lower in males and in newborns delivered by cesarean section (Picone et al., 2012). However, post-mortem infant brain samples analyzed for lutein by HPLC did not show any difference in the levels of lutein between males and females (Lieblein-Boff et al., 2015). Arterial cord blood level of lutein is higher in preterm infants, especially at the early stage of the third trimester, than in term infants. It is possible to speculate that increased value of carotenoids in this period is related to the role in promoting central nervous system development. Lutein and zeaxanthin are found in the eye and are concentrated in the foveal depression of the macula. Previous studies (Vishwanathan et al., 2013) demonstrated a correlation between carotenoid macula concentration and brain value. The measure of total macular pigment may be a tool in investigating the role of lutein in cognitive functions. Retinal lutein is significantly related to lutein level in the occipital cortex. Renzi et al. (2013) found a relationship between macular pigments and visuo-motor response in humans. Carotenoids can enhance gap junctional communication in cell and can ameliorate visual function because gap junctions are crucial to light processing within the retina. Macular pigment optical density correlates with diet intake and with age (Bernstein et al., 2013). Lutein may improve neuroretinal maturation in preterm infants and it is predominant in brain development; these findings confirm that lutein is important for neurodevelopment in the infant but the mechanism is not completely clear (Lieblein-Boff et al., 2015). Post-mortem metabolomic analyses were performed on human infant brain tissues (hippocampus, frontal cortex, occipital cortex) commonly involved in learning and memory to study the interaction between nutrients, lutein, and products of cellular metabolism (e.g., nucleotides, sugars and amino acids; Lieblein-Boff et al., 2015). Fatty acids and amino acid neurotransmitters are positively correlated with lutein in infant brain. Lutein seems to promote maturation of cell membranes and cortical folding and seems to support the oligodendrocyte maturation. Furthermore lutein increases neurotransmitters GABA concentration (Lieblein-Boff et al., 2015). GABA seems to play an important role in modulating neuronal proliferation, maturation and synapse formation (Lieblein-Boff et al., 2015). These findings could explain the effects of carotenoids on cognitive functions and temporal processing speed in young (Lieblein-Boff et al., 2015). Anti-inflammatory, antioxidant and light-absorbing characteristics of carotenoids could be important for neonates especially for preterm babies to prevent the pathogenesis of common diseases of prematurity that may be related to poor neurodevelopmental outcome (e.g., necrotizing enterocolitis, retinopathy of prematurity, bronchopulmonary dysplasia; Rubin et al., 2012). Inflammation generates reactive oxygen species (ROS) that can attack DNA, RNA, lipids and proteins activating various signaling pathways that could cause diseases. In intrauterine life there is very low oxygen concentration but after birth higher levels of oxygen are available for the newborn and ROS as singlet oxygen and hydroxyl radicals are produced (Perrone et al., 2014). Lutein with a hydroxyl group attached to either ends of the molecule can react with singlet oxygen and can neutralize ROS (Figure 1). Oxidative stress (OS), can increase during pregnancy, and it can lead to cellular and tissue damage. Lorenzoni et al. (2013) investigated the oxidative status of diabetic pregnant women and of their babies. Oxidative stress is lower in pregnant women receiving lutein and in their newborns than in untreated mothers and newborns showing that lutein administration during pregnancy significantly reduces neonatal OS at birth. Furthermore infants fed with lutein-supplemented formula seem to develop less severe retinopathy of prematurity (ROP) than control group and they have lower value of plasma C-reactive protein (CRP) (Rubin et al., 2012). These findings may be due to carotenoid immunomodulatory properties (Rubin et al., 2012). Manzoni et al. (2013) in a multicenter, double-blind, randomized controlled trial with lutein supplementation in preterm babies confirmed that threshold ROP incidence tended to be lower in the lutein treated vs. not treated infants. They also found that less severe forms of bronchopulmonary dysplasia (BPD) and necrotizing enterocolitis (NEC) were associated to lutein supplementation. Lutein is a bioactive compound and its potential epigenetic effect on neurodevelopment in preterm infants deserves further studies (Ferguson and Schlothauer, 2012). More studies are necessary to better understand the effects of lutein administration to mothers and newborns and the toxicity of these compounds. If beneficial effects of lutein are confirmed at the tested dosages, this compound should be considered as a potential therapy for the prevention of bronchopulmonary dysplasia, necrotizing enterocolitis and other diseases of prematurity.

**Figure 1:**
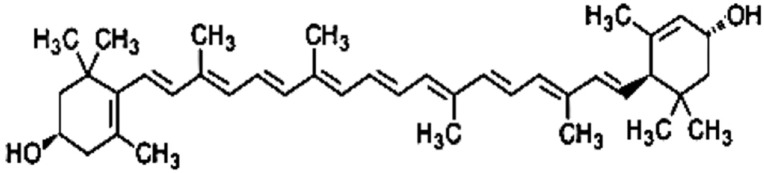
**Lutein**.

## Author contributions

MG wrote the paper. FM, FL searched references. AB, PG supervised the drafting of the paper.

### Conflict of interest statement

The authors declare that the research was conducted in the absence of any commercial or financial relationships that could be construed as a potential conflict of interest. The reviewer GF and handling Editor declared their temporarily shared affiliation, and the handling Editor states that the process nevertheless met the standards of a fair and objective review.
